# The epidemiology and disease burden of children hospitalized for viral infections within the family *Flaviviridae* in China: A national cross-sectional study

**DOI:** 10.1371/journal.pntd.0010562

**Published:** 2022-07-05

**Authors:** Ran Wang, Xinyu Wang, Linlin Zhang, Guoshuang Feng, Mengjia Liu, Yueping Zeng, Zhengde Xie

**Affiliations:** 1 Beijing Key Laboratory of Pediatric Respiratory Infectious Diseases, Key Laboratory of Major Diseases in Children, Ministry of Education, National Clinical Research Center for Respiratory Diseases, Research Unit of Critical Infection in Children, Chinese Academy of Medical Sciences, 2019RU016, Laboratory of Infection and Virology, Beijing Pediatric Research Institute, Beijing Children’s Hospital, Capital Medical University, National Center for Children’s Health, Beijing, China; 2 Big Data Center, Beijing Children’s Hospital, Capital Medical University, National Center for Children’s Health, Beijing, China; 3 Medical Record Management Office, Beijing Children’s Hospital, Capital Medical University, National Center for Children’s Health, Beijing, China; Fundacao Oswaldo Cruz, BRAZIL

## Abstract

**Background:**

Viruses of the family *Flaviviridae*, including Japanese encephalitis virus (JEV), dengue virus (DENV), yellow fever virus (YFV) and hepatitis C virus (HCV), are widely distributed worldwide. JEV, DENV and YFV belong to the genus *Flavivirus*, whereas HCV belongs to the genus *Hepacivirus*. Children’s symptoms are usually severe. As a result, rates of hospitalization due to infection with these viruses are high. The epidemiology and disease burden of hospitalized children have rarely been described in detail to date. The objective of this study was to report the general epidemiological characteristics, clinical phenotype, length of stay (LOS), burden of disease, and potential risk factors for hospitalized children infected with JEV, DENV, YFV, or HCV in Chinese pediatric hospitals.

**Methodology:**

A cross-sectional study of epidemiology and disease burden of children hospitalized for *Flaviviridae* virus infections between December 2015 and December 2020 in China was performed. Face sheets of discharge medical records (FSMRs) were collected from 27 tertiary children’s hospitals in the Futang Research Center of Pediatric Development and aggregated into FUTang Update medical REcords (FUTURE). Information on sociodemographic variables, clinical phenotype, and LOS as well as economic burden was included in FSMRs and compared using appropriate statistical tests.

**Findings:**

The study described 490 children aged 0–15 years hospitalized for infections with *Flaviviridae* viruses. Japanese encephalitis (JE) cases are the highest, accounting for 92.65% of the total hospitalization cases caused by *Flaviviridae* virus infection. The incidence of JE peaked from July to October with a profile of a high proportion of severe cases (68.06%) and low mortality (0.44%). Rural children had a significantly higher incidence than urban children (91.63%). Most hospitalized dengue cases were reported in 2019 when dengue outbreaks occurred in many provinces of China, although only 14 dengue cases were collected during the study period. Yellow fever (YF) is still an imported disease in China. The hospitalizations for children with hepatitis C (HC) were not high, and mild chronic HC was the main clinical phenotype of patients. Among the four viral infections, JE had the highest disease burden (LOS and expenditure) for hospitalized children.

**Conclusion:**

First, the present study reveals that JE remains the most serious disease due to *Flaviviridae* virus infection and threatens children’s health in China. Many pediatric patients have severe illnesses, but their mortality rate is lower, suggesting that existing treatment is effective. Both JEV vaccination and infection control of rural children should represent a focus of study. Second, although the dual risks of indigenous epidemics and imports of DENV still exist, the prevalence of DENV in children is generally manageable. Third, YFV currently shows no evidence of an epidemic in China. Finally, the proportion of children with chronic hepatitis C (CHC) is relatively large among hospitalized children diagnosed with HCV. Thus, early and effective intervention should be offered to children infected with HCV to ease the burden of CHC on public health.

## Author summary

We performed a general epidemiological and disease burden assessment of 490 hospitalized children infected with any virus from the family *Flaviviridae* [Japanese encephalitis virus (JEV), dengue virus (DENV), yellow fever virus (YFV) and hepatitis C virus (HCV)] from December 2015 to December 2020 with confirmed clinical presentation and laboratory results. Our study found that hospitalization for Japanese encephalitis (JE) predominated in children who lived in rural areas, and the infection was rate was considerably higher in summer and autumn (July–October) compared with other months. In addition, children hospitalized with JE have the largest share of disease burden. However, the overall low rate of hospitalization and mortality of children shows that China’s JE prevention and control policies remain effective. However, the prevention, control and surveillance of JEV in rural areas should not be neglected. Dengue and yellow fever have not yet caused serious public health concerns among children in China, but the spatial and temporal distributions of viral infection must be assessed to be alert to the indigenous spread of imported cases. CHC is a refractory phenotype of HCV infection in children; thus, early screening and intervention are encouraged given the insidious appearance of symptoms in the early stages after HCV infection. These findings can help to understand the epidemic status of viruses classified in the family Flaviviridae in children and the disease burden of hospitalized children, which is conducive to precise prevention and control, optimization of the allocation of resources, and the formulation of more reasonable and effective policies.

## Introduction

*Flaviviridae* constitutes a large viral family that includes highly relevant human clinical pathogens. The genera *Flavivirus*, including Japanese encephalitis virus (JEV), dengue virus (DENV), and yellow fever virus (YFV), and the genus *Hepacivirus*, including hepatitis C virus (HCV), are classified into the same family *Flaviviridae*. These viruses comprise a group of small enveloped, positive-stranded RNA viruses that represent global public health problems.

JEV is mainly distributed in Asia, the Western Pacific, and Northern Australia through *Culex* mosquitoes as vectors. Japanese encephalitis (JE) caused by JEV infection is a preventable disease. From the inactivated vaccine, which has been available since the 1950s, to the live attenuated vaccine SA14-14-2, which has already been widely used in China and many Asian countries for many years, the two types of vaccines significantly reduced the incidence of JE. An estimated 67,900 JE cases occur worldwide annually, half of which are located in China [1]. Twenty years ago, approximately 75% of JE patients were children aged 0–14 years, whereas adults were generally considered immune because they had experienced at least one natural infection [2]. The JE fatality rate is 20–30%, and 30–50% of survivors suffer from permanent and irreversible neuropsychiatric sequelae, which have placed a heavy burden on the public health system and society [3]. Therefore, JE has become a public health issue of international concern [4]. According to model estimates, 0.8% to 5.2% of children are naturally infected with JEV each year. However, these findings may be more severe in countries and regions where JEV vaccination is not yet included in the national vaccination program [5]. In China, although a decrease in cases among children has been observed since the Expanded Program on Immunization (EPI) was established in April 2008, sporadic and localized outbreaks are still reported frequently [6]. Correspondingly, children remain an at-risk population [5].

Dengue is one of the most common infectious diseases resulting from mosquito-borne flaviviruses and is also the fastest spreading tropical disease worldwide. DENV is prevalent in southern and central China [7]. There are also some imported cases (indigenous cases that have not been reported in some years) due to the high incidence of dengue in the southern border of China or other tropical and subtropical countries [8, 9]. Dengue has no significant susceptible age, and people of all ages are generally susceptible. Dengvaxia, the only commercial vaccine against dengue, has been licensed in more than 20 endemic countries, but it still faces difficulties given its safety profile and immunogenicity. The WHO’s Strategic Advisory Group of Experts on Immunization (SAGE) is only recommended for people aged 9–45 years old [10]. The vast majority of dengue cases occur in children under the age of 15 years [11], and the clinical spectrum ranges from asymptomatic or mild febrile illness to life-threatening hemorrhagic fever syndrome [12]. Especially in the absence of protective vaccines and targeted treatment, children are at high risk of severe dengue [13].

YFV is the first proven human-pathogenic virus, and it remains a serious public health threat in endemic countries. In contrast to JE and dengue, yellow fever (YF) remains an imported disease in China. On March 12, 2016, the first imported case of YF was confirmed by the Chinese Center for Disease Control and Prevention (CDC). There is no evidence of local transmission of YFV in China to date [14]. Most imported cases originate from Africa and South America, which unmasked the low YF vaccination coverage among Chinese travelers and workers to Africa; however, the effectiveness of the YFV-17D vaccine has been sufficiently confirmed [15]. Descriptions of the disease burden among imported pediatric cases in China are limited.

Hepatitis C (HC), an important infection-induced inflammatory liver disease, is caused by HCV (family *Flaviviridae*, genus *Hepacivirus*) transmission through the blood. The disease is extremely insidious and progresses slowly. Eighty percent of infected individuals can progress to chronic hepatitis C (CHC), and some patients may further develop cirrhosis or even liver cancer if timely treatment is not provided. HC is now classified as a category B notifiable infectious disease in China [16]. According to the World Health Organization (WHO) report, approximately 250 million people worldwide are infected with HCV, of which 3.26 million are children (0–18 years old) [17]. The prevalence of HC increases with age. China is a country with a moderate disease burden for HC [18], and the incidence rate of HCV infection related to transfusion or blood products (invasive medical procedures) has been dramatically reduced due to the implementation of mandatory blood transfusion screening of transfusion recipients since 1993 [19]. In addition to transfusion, 4–10% of HCV infections can be acquired via mother-to-fetus transmission, which is also the most common transmission route of acquisition for children [20]. In contrast, transmission through casual contact is infrequent [21]. Most infants can clear HCV spontaneously. HCV infection in patients during childhood is typically asymptomatic and slowly progressing, and serious complications rarely occur [22]. Nevertheless, HCV infection acquired through vertical transmission in children still has the chance to develop into CHC, end-stage liver disease, and even hepatocellular carcinoma in adulthood without efficient treatment. At present, direct-acting antivirals (DAAs) are an effective method to cure HCV infection [23, 24]. The disease burden of HCV infection in adults and at-risk populations has been fully reported, whereas the available data on the pediatric burden of HC are limited.

Children infected with the aforementioned *Flaviviridae* virus are more likely to be hospitalized than adults. However, hospitalized children’s epidemiological and economic burdens have not been described in detail. The electronic medical system of Chinese hospitals has generated a huge amount of medical data. The massive amounts generated during the patient’s hospitalization are mainly unstructured data; among these, face sheets of discharge medical records (FSMRs) comprise big data with the highest degree of standardization, the easiest feature for data-mining, and the most evidence-based medicine value. This study is based on a national pediatric patient database of China, including multicenter, observational, cross-sectional studies of 27 pediatric hospitals, to ensure geographic representation and reduce bias to truthfully and objectively describe and analyze the most common epidemiological characteristics, clinical phenotype, length of stay (LOS), expense (in US dollar terms, USD) and potential risk factors for hospitalized children infected with any virus within *Flaviviridae* (JEV, DENV, YFV and HCV) in Chinese pediatric hospitals. The data obtained can provide important support for the evidence-based Chinese pediatric *Flaviviridae* virus infection and the prevention policy and strategy for the pediatric population and help evaluate the effectiveness of the prevention and treatment of infection with the virus of the family *Flaviviridae* in the Chinese population.

## Methods

### Study area

This study is located in the People’s Republic of China (Mainland China), which is located on the east coast of Asia and the west coast of the Pacific Ocean. The latitude spanning north and south of China’s territory is approximately 50°, most of which is in the temperate zone and a small part in the tropics. According to data released by the National Bureau of Statistics of the People’s Republic of China on January 17, 2020, the total population of mainland China is 1.45 billion, ranking first in the world. Of these individuals, approximately 250 million are children.

### Data source

The Futang Research Center of Pediatric Development (FRCPD) is the first nonprofit social service organization engaged in pediatric development research under the supervision and management of the Ministry of Civil Affairs of China. The FRCPD has established a network of health services by integrating 47 provincial children’s medical institutions (approximately 77% of the national public children’s hospitals) as the core. The data of this study are retrospectively extracted from FUTang Update medical REcords (FUTURE) [25], covering the FSMRs of hospitalized children in 27 tertiary children’s hospitals in FRCPD (21 of them are located in the provincial capital). The FRCPD started to collect FSMRs data in December 2015, and the staff at FRCPD were responsible for checking and validating the uploaded data to control its quality and integrity [25].

### Study variables

The variables collected by the FSMRs include sociodemographic and geographic variables, admission and discharge information, primary and secondary diagnoses and hospitalization expenses of the patients. We reported the epidemiology of the cohort based on the type of virus infection and stratified the cohort based on gender, age groups, geographic regions, ethnicity, and complications. Among them, hospitalized patients are divided into five groups according to their age (day, d, or year, y): 0–28 d (neonate), 29 d–<1 y (infant), 1–3 y (toddler), 4–6 y (preschool child), and 7–15 y (school-age child). According to the diagnostic criteria promulgated by the Health Commission of the People’s Republic of China (formerly the Ministry of Health), the clinical manifestation of JE is generally classified into mild, moderate, severe and critical (http://www.nhc.gov.cn/wjw/s9491/200907/41978.shtml); dengue is clinically defined as mild and severe (http://www.nhc.gov.cn/wjw/s9491/201803/d524df26df28453eada8371dc3565818.shtml); and HC is clinically divided into acute icteric, acute anicteric, mild chronic, and moderate chronic categories (http://www.nhc.gov.cn/wjw/s9491/201803/29997c16d2f24e639ab6c6f55105a9d0.shtml). The 27 children’s hospitals are grouped into seven geographic regions: northeast, north, east, south, central, northwest, and southwest China (**Table 1**).

**Table 1:** The geographic distribution of 27 children’s hospitals in the FUTang Updating medical REcords (FUTURE) database.

Region	Hospitals in FUTURE database
Northeast China	Changchun Children’s Hospital Liaoning Children’s Hospital Dalian Women and Children’s Medical Group
North China	Beijing Children’s Hospital, Capital Medical University Baoding Children’s Hospital Children’s Hospital of Hebei Province Children’s Hospital of Shanxi Inner Mongolia Maternity and Child Health Care Hospital
East China	Children’s Hospital Affiliated to Shandong University Women and Children Hospital in Liaocheng Children’s Hospital of Nanjing Medical University Children’s Hospital of Soochow University Hangzhou Children’s Hospital Fuzhou Children’s Hospital of Fujian Province Anhui Provincial Children’s Hospital Jiangxi Provincial Children’s Hospital
South China	Liuzhou Maternity and Child Healthcare Hospital Shenzhen Children’s Hospital
Northwest China	Gansu Provincial Maternity and Child-care Hospital Urumqi Children’s Hospital Qinghai Province Women and Children’s Hospital Xi’an Children’s Hospital
Southwest China	Guiyang Children’s Hospital Kunming Children’s Hospital
Central China	Henan Children’s Hospital Hunan Children’s Hospital Wuhan Children’s Hospital, Tongji Medical College, Huazhong University of Science & Technology

### The eligibility of the participants and admission records

The inclusion criteria of the participants were as follows:

1. All included patients who were admitted with the primary diagnosis of any infection of the family *Flaviviridae* between December 1^st^, 2015 and December 31^st^, 2020. The 10^th^ Revision of International Statistical Classification of Diseases and Related Health Problems (WHO, ICD-10) codes (https://icd.who.int/browse10/2016/en) was used as the selection criteria for the primary screening and classification of disease.
2. All included patients had a clear pathogenic diagnosis (serological IgM antibody testing).

The exclusion criteria of the participants were as follows:

1. Data with multiple vital data incomplete, such as patient sex, age, diagnosis, or burden of disease (LOS and expense), were excluded.
2. The patients were aged ≤18 years on the index admission.

### Statistical analysis

Categorical variables are described as percentages. The median with interquartile ranges (IQR) is used as a measure of central tendency. For nonordinal categorical variables, the chi-squared test was used to compare percentages among groups. For ordinal categorical variables and nonnormal distribution data, the nonparametric Wilcoxon or Kruskal–Wallis test was used for the comparison among groups. All statistics were analyzed by using SPSS software version 22.0 (SPSS Inc., USA). Differences with p values of < 0.05 were considered statistically significant.

### Ethics statement

This study was approved by the Ethics Committee of Beijing Children’s Hospital, Capital Medical University (Approval Number: [2021]-E-209-R). The requirement for informed consent from patients was waived given that the study exclusively involved a retrospective aggregated data analysis of medical records. Our data are fully deidentified and anonymous to protect privacy.

## Results

### Overall

We collected 5,790,465 FSMRs of hospitalized patients under the framework of FRCPD. According to the ICD-10 codes, we searched for hospitalizations with a clear diagnosis of infections with any virus within the family *Flaviviridae* (JEV, DENV, YFV and HCV). As **Table 2** shows, a total of 490 patients were hospitalized for infection with any one of the above four viruses. Among infections with any genus *Flavivirus*, JE cases accounted for the largest proportion (454 cases; 92.65%) followed by dengue (14 cases, 2.86%) and three imported yellow fever cases (0.61%). For the *Hepacivirus* genus, only 19 hospitalized cases with HCV infection were reported (3.88%). However, there was only one neonatal case of HCV, and none of the cases aged 16–18 years had been reported in all the cases. Hospitalized children with JE had the longest LOS of 15 (11–24) days and the highest expenditure of 7,504.79 USD (IQR 15,247.22–32,179.74), indicating that this disease burden was the highest.

**Table 2:** The general sociodemographic characteristics of *Flaviviridae* virus infections during pediatric hospitalizations from December 2015 to December 2020.

*Flaviviridae* virus				
Categories	*Flavivirus*			*Hepacivirus*
	JEV	DENV	YFV	HCV
**Overall (n, %)**	454 (92.65)	14 (2.86)	3 (0.61)	19 (3.88)
**Gender (n, %)**				
Male	266 (58.59)	7 (50.00)	2 (66.67)	11 (57.89)
Female	188 (41.41)	7 (50.00)	1 (33.33)	8 (42.11)
**Age (n, %)**				
0–28 d	0 (0.00)	0 (0.00)	0 (0.00)	1 (5.26)
29 d–<1y	4 (0.88)	0 (0.00)	0 (0.00)	1 (5.26)
1–3 y	154 (33.92)	2 (14.29)	1 (33.33)	8 (42.11)
4–6 y	75 (16.52)	5 (35.71)	1 (33.33)	7 (36.84)
7–15 y	221 (48.68)	7 (50.00)	1 (33.33)	2 (10.53)
**Region (n, %)**				
Northeast China	0 (0.00)	1 (7.14)	0 (0.00)	0 (0.00)
North China	4 (0.88)	0 (0.00)	0 (0.00)	0 (0.00)
East China	294 (64.76)	5 (35.71)	0 (0.00)	0 (0.00)
South China	0 (0.00)	3 (21.43)	0 (0.00)	2 (10.53)
Northwest China	29 (6.39)	0 (0.00)	1 (33.33)	9 (47.37)
Southwest China	35 (7.71)	3 (21.43)	0 (0.00)	1 (5.26)
Central China	92 (20.26)	2 (14.29)	2 (66.67)	7 (36.84)
**Ethnicity (n, %)**				
Han	436 (96.04)	13 (94.74)	3 (100.00)	18 (94.74)
Non-Han	18 (3.96)	1 (5.26)	0 (0.00)	1 (5.26)
**Year of admission (n, %)**				
2015^1^	1 (0.22)	0 (0.00)	0 (0.00)	0 (0.00)
2016	153 (33.70)	2 (14.29)	0 (0.00)	1 (5.26)
2017	118 (25.99)	0 (0.00)	1 (33.33)	2 (10.53)
2018	119 (26.21)	1 (7.14)	0 (0.00)	3 (15.79)
2019	34 (7.49)	11 (78.57)	2 (66.67)	7 (36.84)
2020	29 (6.39)	0 (0.00)	0 (0.00)	6 (31.58)
**LOS [d, median (IQR)]** ^3^	15 (11–24)	4 (2.75–6.25)	3 (3–4)	4 (2–6)
**Expense [USD, median (IQR)]** ^4^	7,504.79 (15,247.22–32,179.74)	5,058.19 (779.26–9,961.71)^2^	6,895.65 (5,815.71–19,387.27)	3,460.41 (2,681.17–5,598.76)

JEV: Japanese encephalitis virus

DENV: dengue virus

YFV: yellow fever virus

HCV: hepatitis C virus

LOS: length of stay

IQR: inter quartile range

USD: USA dollar

^1^Only the data for December 2015 is presented

^2^Data deficient of two children

^3^Kruskal-Wallis test, whole: *Z* = 69.906, p<0.0001; Mann-Whitney-Wilcoxon test, JEV *vs*. DENV: *Z* = 5.225, p<0.0001, JEV *vs*. YFV: *Z* = 2.735, p = 0.03, JEV *vs*. HCV: *Z* = 6.175, p<0.0001

^4^Kruskal-Wallis test, whole: *Z* = 52.279, p<0.0001; Mann-Whitney-Wilcoxon test, JEV *vs*. DENV: *Z* = 4.018, p = 0.0003, JEV *vs*. HCV: *Z* = 6.127, p<0.0001

### JEV

The male to female ratio of JEV-infected children was 1.41:1. School-age children (7–15 y; 48.68%) and toddlers (1–3 y; 33.92%) accounted for greater than 80%, and neonatal patients (0–28 d) and young (16–18 y) patients were unreported in the age group. The highest hospitalization rate for JE was observed in east China, accounting for 64.76% of the total hospitalized cases, followed by central China (20.26%), southwest China (7.71%), northwest China (6.39%), and north China (0.88%). No hospitalized cases have been reported in northeast and south China. Hospitalization was dominant in the Han population (96.04%). The number of hospitalizations in 2016–2018 was relatively high, accounting for 33.70%, 25.99%, and 26.21% of the total, respectively. However, hospitalizations declined in 2019 and 2020 (only data in December were involved in 2015) (**Table 2**).

**Fig 1:**
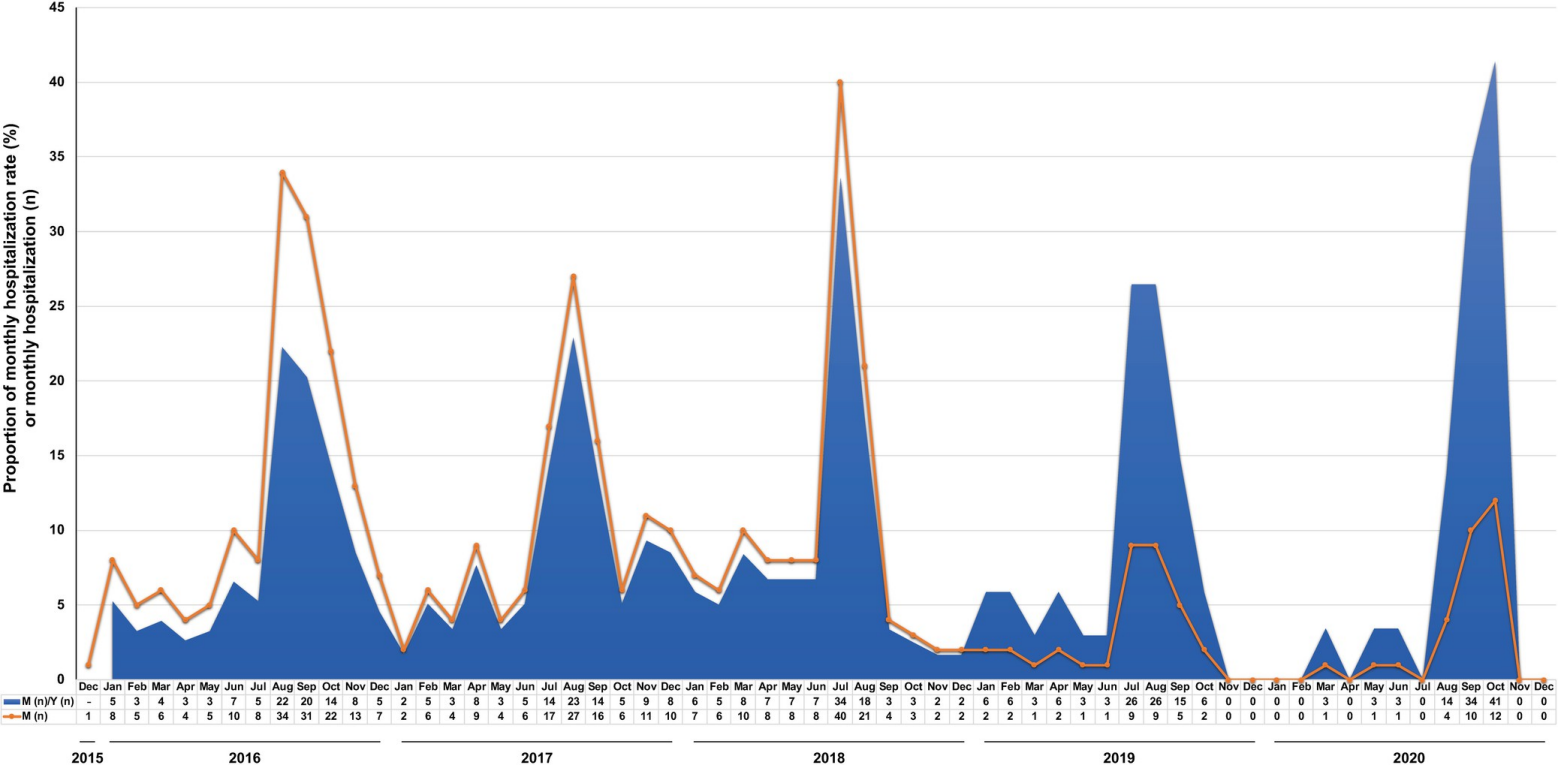
Hospitalization rate and number of JEV infections (*n* = 454), December 2015–December 2020. M (n): number of monthly hospitalizations, Y (n): number of yearly hospitalizations.

The presented FSMR data demonstrate a seasonality in the prevalence of JEV. The peak number of cases and hospitalization rates were from August to October in both 2016 and 2020 and from July to September in 2017 and 2019, whereas the statistics in July and August were mainly higher during 2018. Therefore, the high incidence of JE typically occurs in summer and autumn (**Fig 1**).

Children with severe symptoms were the main clinical phenotype of severity, accounting for 68.06% of cases. Moderate (14.76%), mild (12.11%) and critical (5.07%) cases together accounted for greater than 30% of patients (**Table 3**). The main clinical type of children was severe regardless of subgroup analysis based on sex, age group, the five regions where cases were reported, ethnicity, residence (urban or rural), and year of admission. In detail, the 1–3 y group had more severe clinical symptoms than the 7–15 y group (z = 27.135, p = 0.00001), and the children living in urban areas were more severe than those in rural areas (z = 5.561, p = 0.018). Children who were hospitalized in 2017 were more severe than those who were hospitalized in 2018 (z = -3.033, p = 0.03). Although children with different clinical categories had similar LOSs (z = 1.724, p = 0.632) and the median was approximately 15 days, the economic burden of severe children was the lowest (z = 61.347, p<0.0001) with a median of only 1,641.56 USD (IQR 1,019.19–3,804.88). The expenditure of severe JE is also relatively low on average, which is consistent with trends noted for the median.

**Table 3:** The general sociodemographic characteristics and disease burden of JEV infection categorized by clinical classification during pediatric hospitalizations from December 2015 to December 2020.

Categories	Clinical Classification				*Z* value	p value
	Mild	Moderate	Severe	Critical		
**Overall (n, %)**	55 (12.11)	67 (14.76)	309 (68.06)	23 (5.07)	-	-
**Gender (n, %)**						
Male	37 (13.91)	44 (16.54)	188 (62.78)	18 (6.77)	1.171	0.241
Female	18 (9.57)	23 (12.23)	143 (75.53)	5 (2.66)		
**Age (n, %)**						
29 d–<1y	0 (0.00)	0 (0.00)	4 (100.00)	0 (0.00)	22.278	0.0004^2^
1–3 y	5 (3.25)	17 (11.04)	126 (81.82)	6 (3.90)		
4–6 y	11 (14.67)	9 (12.00)	50 (66.67)	5 (6.67)		
7–15 y	39 (17.65)	41 (18.55)	129 (58.37)	12 (5.43)		
**Region (n, %)**						
North China	0 (0.00)	0 (0.00)	4 (100.00)	0 (0.00)	9.675	0.05
East China	34 (11.56)	31 (10.54)	224 (76.19)	5 (1.70)		
Northwest China	4 (13.79)	10 (34.48)	13 (44.83)	2 (6.90)		
Southwest China	5 (14.29)	11 (31.43)	17 (48.57)	2 (5.71)		
Central China	12 (13.04)	15 (16.30)	51 (55.43)	14 (15.22)		
**Ethnicity (n, %)**						
Han	55 (12.61)	62 (14.22)	297 (68.12)	228 (5.05)	0.259	0.796
Non-Han	0 (0.00)	5 (27.78)	12 (66.67)	1 (5.56)		
**Residence (n, %)**						
Urban	2 (5.26)	2 (5.26)	31 (81.58)	3 (7.89)	5.561	0.018
Rural	53 (12.74)	65 (15.63)	278 (66.83)	20 (4.81)		
**Year of admission (n, %)**						
2015^1^	0 (0.00)	0 (0.00)	1 (100.00)	0 (0.00)	11.905	0.04^3^
2016	19 (12.42)	18 (11.76)	109 (71.24)	7 (4.58)		
2017	5 (4.24)	16 (13.56)	91 (77.12)	6 (5.08)		
2018	20 (16.81)	24 (20.17)	68 (57.14)	7 (5.88)		
2019	4 (11.76)	5 (14.71)	23 (67.65)	2 (5.88)		
2020	7 (24.14)	4 (13.79)	17 (58.62)	1 (3.45)		
**LOS [d, median (IQR)]**	15 (12–24)	17 (13–22)	15 (11–25)	15 (14–18)	1.724	0.632
**Expense [USD, median (IQR)]**	3,868.73 (3,474.44–5,776.05)	4,657.10 (2,965.60–6,490.27)	1,641.56 (1,019.19–3,804.88)	4,313.48 (1,745.83–9,782.99)	61.347	<0.0001

JEV: Japanese encephalitis virus

LOS: length of stay

IQR: inter quartile range

USD: USA dollar

^1^Only the data for December 2015 is presented

^2^1–3 y group *vs*. 7–15 y group, *Z* = -4.214, p = 0.0001

^3^2017 group *vs*. 2018 group, *Z* = -3.033, p = 0.03

The results showed that 91.63% of children with JE lived in rural areas, whereas only 8.37% of children lived in urban areas. Similarly, regardless of sex, age, region, ethnicity, and year of admission, the hospitalization proportion of rural children was absolutely dominant, but no significant differences were noted among the subgroups (**Table 4**). LOS and expenses were also not significantly different between children in urban and rural areas (**Table 4**).

**Table 4:** The general sociodemographic characteristics and disease burden of JEV infection categorized by residence during pediatric hospitalizations from December 2015 to December 2020.

Categories	Residence		*χ*^2^*/Z* value	p value
	Urban	Rural		
**Overall (n, %)**	38 (8.37)	416 (91.63)	-	-
**Gender (n, %)**				
Male	25 (9.40)	241 (90.60)	0.886	0.347
Female	13 (6.91)	175 (93.09)		
**Age (n, %)**				
29 d–<1y	1 (25.00)	3 (75.00)	6.719	0.081
1–3 y	15 (9.74)	139 (90.26)		
4–6 y	10 (13.33)	65 (86.67)		
7–15 y	12 (5.43)	209 (94.57)		
**Region (n, %)**				
North China	0 (0.00)	4 (100.00)	5.514	0.239
East China	31 (10.54)	263 (89.46)		
Northwest China	1 (3.45)	28 (96.55)		
Southwest China	1 (2.86)	34 (97.14)		
Central China	5 (5.43)	87 (94.57)		
**Ethnicity (n, %)**				
Han	36 (8.26)	400 (91.74)	0.184	0.668
Non-Han	2 (11.11)	16 (88.89)		
**Year of admission (n, %)**				
2015^1^	0 (0.00)	1 (100.00)	5.595	0.348
2016	14 (9.15)	139 (90.85)		
2017	11 (9.32)	107 (90.68)		
2018	7 (5.88)	112 (94.12)		
2019	1 (2.94)	33 (97.06)		
2020	5 (17.24)	24 (82.76)		
**LOS [d, median (IQR)]** ^2^	14 (9.75–26.25)	15 (11–23)	-0.843	0.399
**Expense [USD, median (IQR)]** ^2^	1,759.95 (1,014.13–5,309.16)	2,461.74 (1,213.49–5,030.98)	-1.167	0.243

JEV: Japanese encephalitis virus

LOS: length of stay

IQR: inter quartile range

USD: USA dollar

^1^Only the data for December 2015 is presented

^2^Kruskal-Wallis test

**Fig 2:**
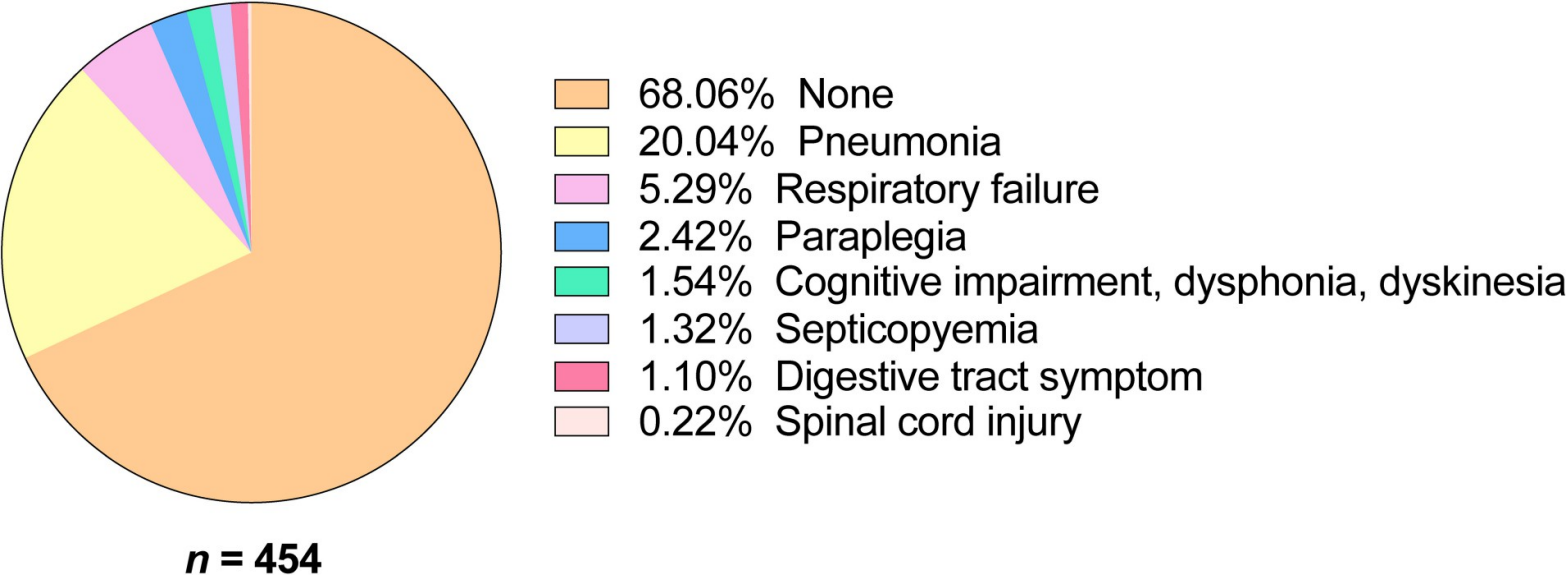
Distribution of complications in hospitalized children with JEV infection (*n* = 454).

Among the 454 hospitalized children, 68.06% had no significant complications except for the symptoms caused by JE itself (**Fig 2**). Pneumonia was the main complication of JE with a rate of 20.04%. Respiratory failure, paraplegia, cognitive impairment/dysphonia/dyskinesia, septicopyemia, digestive tract symptoms, and spinal cord injury accounted for 5.29%, 2.42%, 1.54%, 1.32%, 1.10%, and 0.22% of cases, respectively (**Fig 2**).

Two deaths due to JE were reported in FSMRs. The two patients were both from Anhui Province (East China), lived in rural areas and were clinically diagnosed as critical. These patients were complicated by brain hernia and encephaledema, respectively. The overall mortality rate was only 0.44%. Other sociodemographic characteristics and burden of disease are shown in **Table 5.**

**Table 5:** The general sociodemographic characteristics and disease burden of two deaths from JEV infection during pediatric hospitalizations from December 2015 to December 2020.

No.	Gender	Age (years)	Province (Region)	Ethnicity	Residence	Admission time	Clinical Classification	Cause of death	LOS (d)	Expense (USD)
1	Male	10	Anhui (East China)	Han	Rural	August, 2016	Critical	Brain hernia	14	12,435.24
2	Female	8	Anhui (East China)	Han	Rural	September, 2016	Critical	Encephaledema	9	6,610.59

JEV: Japanese encephalitis virus

LOS: length of stay

USD: USA dollar

### DENV

The dual risk of both imported and indigenous epidemics places China at risk for dengue. The 14 DENV-infected children reported in FSMRs were not classified as either imported or indigenous cases. These children were mainly distributed in the southeastern coastal provinces and south and central China. No cases have been reported in north and northwest China, which is consistent with the prevalence of DENV in China. There was no significant preference for sex, age (no cases under 1 year old), or ethnicity (**Table 2**). Although both DENV and JEV are transmitted through mosquito vectors, the residence of the 14 cases of children did not show a higher incidence in rural areas than that in the children with JE. A total of 78.57% of the cases were hospitalized in 2019. Mild cases include fever (dengue fever), headache, arthralgia and myalgia, and rashes, whereas severe cases include dengue hemorrhagic fever and dengue shock syndrome. The vast majority (92.86%) of the cases reported in this study were mild, and only one severe case complicated with hemophagocytic lymphohistiocytosis was diagnosed. No deaths were noted among the 14 cases. The median LOS of the children was 4 days (IQR 3–6), and the median expense was 1,013.56 USD (IQR 692.17–1,487.60) (**Table 6**).

**Table 6:** The general sociodemographic characteristics and disease burden of DENV infection during pediatric hospitalizations from December 2015 to December 2020.

No.	Gender	Age (years)	Province (Region)	Ethnicity	Residence	Admission time	Clinical Classification	Complication	LOS (d)	Expense (USD)
**1**	Female	1	Fujian (East China)	Han	Rural	August, 2016	Mild	None	3	-^1^
**2**	Male	4	Fujian (East China)	Han	Rural	Sepember, 2016	Mild	None	2	-^1^
**3**	Female	12	Yunnan (Southwest China)	Han	Urban	August, 2018	Mild	None	2	1,646.40
**4**	Male	6	Yunnan (Southwest China)	Han	Rural	August, 2019	Mild	None	6	1,378.29
**5**	Male	12	Zhejiang (East China)	Han	Urban	August, 2019	Mild	None	3	508.39
**6**	Male	3	Hunan (Centeal China)	Han	Rural	Sepember, 2019	Mild	None	3	358.73
**7**	Female	4	Zhejiang (East China)	Han	Urban	Sepember, 2019	Mild	None	4	880.06
**8**	Male	7	Guangdong (South China)	Han	Urban	Sepember, 2019	Mild	None	5	3,065.97
**9**	Female	8	Yunnan (Southwest China)	Han	Rural	Sepember, 2019	Mild	None	17	2,315.36
**10**	Female	12	Guangdong (South China)	Han	Rural	October, 2019	Mild	None	4	1,434.67
**11**	Female	8	Guangdong (South China)	Non-Han	Rural	October, 2019	Mild	None	6	1,147.06
**12**	Male	5	Hunan (Centeal China)	Han	Rural	October, 2019	Mild	None	7	775.64
**13**	Female	5	Yunnan (Southwest China)	Han	Urban	October, 2019	Mild	None	8	716.93
**14**	Male	10	Jiangxi (East China)	Han	Urban	December, 2019	Severe	Hemophagocytic lymphohistiocytosis	1	617.88
**Median (IQR)**	-	4.25 (6.5–9.5)	-	-	-	-	-	-	4 (3–6)	1,013.56 (692.17–1,487.60)

DENV: dengue virus

LOS: length of stay

IQR: inter quartile range

USD: USA dollar

^1^Data deficient

### YFV

Since the first case of YFV infection was reported in Beijing, China in March 2016, all cases have been imported cases to date. This study reported three imported cases of YFV included in FRCPD information acquisition and management. One case was from Beijing (1 year old, male, Han nationality, 2017), and the other two cases were from Hunan (15 years old, male and 4 years old, female, 2019). The symptoms of the three patients were mild with only fever, myalgia, headache, and chills without other complications. The LOS was 3–4 days, and the expense was between 912.48 and 3,041.86 USD (**Table 7**).

**Table 7:** The general sociodemographic characteristics and disease burden of imported YFV infection during pediatric inpatient hospitalizations from December 2015 to December 2020.

No.	Gender	Age (years)	Province (Region)	Ethnicity	Admission time	Complication	LOS (d)	Expense (USD)
**1**	Male	1	Beijing (North China)	Han	May, 2017	None	3	3,041.86
**2**	Male	15	Hunan (CentralChina)	Han	January, 2019	None	4	1,081.93
**3**	Female	4	Hunan (Central China)	Han	January, 2019	None	3	912.48

YFV: yellow fever virus

LOS: length of stay

USD: USA dollar

### HCV

A total of 19 children with HC were reported in this study. The male to female ratio was 1.38:1. The 1–3 y group and the 4–6 y group accounted for 42.11% and 36.84%, respectively. Other age groups shared 21.05% of cases, and the median age was 3 years (IQR 1–4.5). Cases were distributed in northwest (47.37%), central (36.84%), south (10.53%), and southwest China (5.26%). The Han population accounted for 94.74% of cases, and all cases were reported from 2016 to 2020 (**Table 2** and **Table 8**). The 19 children mainly experienced mild chronic HC (57.89%) followed by acute anicteric (31.58%), acute icteric (5.26%), and moderate chronic (5.26%). Only one child who was clinically defined as having acute icteric HC had myocardial damage. The median LOS and expense of these children were 4 days (IQR 2.5–6) and 542.94 USD (IQR 452.88–822.03), respectively (**Table 8**).

**Table 8:** The general sociodemographic characteristics and disease burden of HCV infection during pediatric hospitalizations from December 2015 to December 2020.

No.	Gender	Age (years)	Province (Region)	Ethnicity	Admission time	Clinical Classification	Complication	LOS (d)	Expense (USD)
**1**	Female	5	Yunnan (Southwest China)	Han	July, 2016	Acute anicteric	None	3	343.81
**2**	Male	0^1^	Hubei (Centtral China)	Non-Han	March, 2017	Mild chronic	None	2	611.70
**3**	Female	1	Hubei (Centtral China)	Han	July, 2017	Mild chronic	None	2	485.09
**4**	Male	7	Shaanxi (Northwest China)	Han	January, 2018	Mild chronic	None	6	765.62
**5**	Male	6	Guangdong (South China)	Han	March, 2018	Acute anicteric	None	6	711.26
**6**	Male	4	Shaanxi (Northwest China)	Han	March, 2018	Acute icteric	Myocardial damage	15	1,894.94
**7**	Female	9	Hubei (Centtral China)	Han	July, 2019	Acute anicteric	None	2	523.40
**8**	Female	6	Hunan (Centtral China)	Han	April, 2019	Mild chronic	None	10	911.98
**9**	Male	1	Guangxi (South China)	Han	May, 2019	Acute anicteric	None	4	542.94
**10**	Female	1	Shaanxi (Northwest China)	Han	May, 2019	Acute anicteric	None	5	650.07
**11**	Female	1	Hunan (Centtral China)	Han	July, 2019	Moderate chronic	None	6	500.62
**12**	Male	3	Shaanxi (Northwest China)	Han	November, 2019	Mild chronic	None	10	878.45
**13**	Male	3	Shaanxi (Northwest China)	Han	December, 2019	Mild chronic	None	2	328.90
**14**	Male	4	Shaanxi (Northwest China)	Han	January, 2020	Mild chronic	None	1	120.20
**15**	Male	4	Shaanxi (Northwest China)	Han	April, 2020	Mild chronic	None	3	541.27
**16**	Male	2	Hunan (Centtral China)	Han	June, 2020	Mild chronic	None	4	1,146.16
**17**	Female	1	Hunan (Centtral China)	Han	June, 2020	Mild chronic	None	5	1,240.02
**18**	Male	4	Shaanxi (Northwest China)	Han	July, 2020	Mild chronic	None	3	420.68
**19**	Female	0^1^	Shaanxi (Northwest China)	Han	July, 2020	Acute anicteric	None	6	401.13
**Median (IQR)**	-	3 (1–4.5)	-	-	-	-	-	4 (2.5–6)	542.94 (452.88–822.03)

HCV: hepatitis C virus

LOS: length of stay

IQR: inter quartile range

USD: USA dollar

^1^29 d–<1y

## Discussion

The data of this study show that JEV is the virus within the family *Flaviviridae* that poses the greatest threat to children’s health and leads to the heaviest disease burden among the four viruses in mainland China. In 2004, the WHO report described that JE had overtaken dengue fever as the most important arbovirus disease with a global disease burden [26]. The symptoms, disability, and even death caused by JEV infection are more serious than those of any other arthropod-borne virus. Moreover, no specific treatment is currently available.

Regarding the epidemiological characteristics of JE, our research is consistent with mainstream findings.

First, JE mainly occurs in children under 14 years old [27], and the age of the at-risk population presents an increasing tendency [5, 28]. The results of this study showed that children aged 7–15 years accounted for approximately half of the total number of hospitalizations, which might also be related to the waning positive rate of neutralizing antibodies with age. Previous papers noted that even for children who were completely vaccinated with two shots of JE vaccines, only 25% of vaccinees were positive for JEV neutralizing antibodies (nAbs) at 7.5–8.5 years (age 9.5–11.5 years) after vaccination, and the titer was low [28]. The nAbs continued to decline to lower levels in adulthood [29]. Thus, the use of the third booster shot (the fourth to fifth booster shots of inactivated vaccines) before the age of 18 may further increase the titers of nAbs and should be taken into consideration in policy formulation. The illnesses of children aged 1–3 years were more serious than those of older patients. As previously reported, children with immunocompromised systems had severe clinical symptoms and sequelae [30, 31].

Second, JEV is prevalent in all regions of China. Currently, only Xinjiang, Tibet, and Qinghai have not included JE vaccination in the provincial EPI. The sentinel hospitals covered by the FUTURE database are spread across seven geographic regions. Hospitalized cases have been reported in east, central, southwest, northwest, and north China, of which east China reported the largest number of hospitalized cases. JE remains a disease with high incidence in rural areas, although sporadic cases have been reported in cities and suburbs [32]. In general, JEV is more likely to spread in environments with poor hygiene, such as rural areas, where mosquitoes and swine are more numerous. Therefore, we described the epidemiology and disease burden of hospitalized children based on the place of residence [33].

Third, most JE patients were asymptomatic or had mild [34]; thus, the hospitalized cases included patients with more severe symptoms. Our data showed that patients with severe symptoms were predominant (68.06%). In addition, we also determined for the first time that the main complications of hospitalized children with JE were pneumonia and respiratory failure, and the causes of death included brain hernia and encephaledema.

Fourth, the economic burden assessed in this study was derived from data-directed medical costs from hospitals’ billing systems, i.e., inpatient costs (including patient out-of-pocket medical costs). In general, the number of hospitalizations, hospitalization rates, and mortality of children with JE have significantly improved, suggesting that the existing prevention and control policies and treatment methods are effective. There are few reports on the burden of JE disease in children, especially research on age restratification. We calculated a median LOS of 15 days and costs of 7,504.79 USD in this study. In a longitudinal comparison, children who were clinically defined as having severe JE have a lower expense. It is hypothesized that the largest number of children with severe symptoms and the longest span of hospitalization expenditure may set the median apart from other categories where the numbers of cases are far less than severe JE. However, the real reason needs to be explored in depth. In addition, in the horizontal comparison of the four viruses within the family *Flaviviridae*, JEV infection caused the longest hospitalization LOS and the highest economic burden, indicating that the disease burden caused by JE on children remains the greatest among several common infections with viruses within the family *Flaviviridae* in mainland China. Nevertheless, the data suggest the importance of JE vaccination, routine surveillance, and infection control in rural children.

Since 1978, dengue has become indigenous in China every 4–7 years with a high incidence in people between 20 and 60 years old. The incidence in males and females was similar with mild disease predominantly noted [35]. The dengue epidemic in China is not only indigenous but also imported, which is similar to JE, but there are still more cases of indigenous people than individuals [36, 37]. Imported dengue cases that occur in waves in southeast Asia can also be secondary to indigenous dengue fever in different regions of China [9]. Due to geographical, topographic and climatic characteristics, the majority of dengue cases in China are concentrated along the southwest border of Yunnan and through the Pearl River Delta [38]. In 2019, a dengue outbreak occurred in mainland China, affecting 13 provinces with a total of 15,187 indigenous cases. The number of cases in this epidemic was more than the annual number of cases during 2005–2018 (except for 2014) [39]. The spatial distribution of indigenous cases was not only located in the provinces with occasional reports on the southwest border, such as Yunnan, Guangdong, Fujian, Zhejiang, South China, and the southeast coast [40–42] but also in provinces with previously few reports, such as Hunan, Jiangxi, and Chongqing [39]. Our statistics showed that 78.57% of cases were hospitalized in 2019, and most of these cases were distributed in the southeastern coastal region and south and central China. The spatial and temporal patterns and clinical features are consistent with the epidemiological background.

Markedly, most cases were found from August to October, although the hospitalizations were limited, which corresponds with the temporal feature of the peak period of indigenous incidence (September and October) [41]. However, the low hospitalization rate induced by dengue suggests that the impact of the dengue epidemic on children remains under control in China. Furthermore, we also present the disease burden of hospitalized children with dengue. Nevertheless, given the limited number of cases, it is difficult to discuss the impact of factors, such as age, gender, ethnicity, and residence, on disease burden.

As the first proven human-pathogenic virus, YFV remains a major public health problem, and an upsurge was recently noted. Fortunately, the YFV-17D vaccine still has protective immunity against circulating strains [43]. This article summarizes the relevant information and disease burden of three confirmed YF cases imported from Africa into China, highlighting the potential transmission of YFV through Asia. Nevertheless, YF is an imported disease [44]; hence, the potential and ever-present public health threat to children caused by international exchanges should not be ignored.

HC is the only viral infectious disease of the genus *hepacivirus* in this study. HCV infection has become the second-largest type of viral liver disease in China after hepatitis B virus infection [45], posing a huge threat to public health. Children are susceptible to HCV without a gender preference. However, due to transfusion and blood product control, most children with HCV are infected by vertical transmission with a probability of ~5% [46]. Forty percent of pediatric patients who acquire HCV through vertical transmission can clear the virus spontaneously, even in the absence of special treatment until they are 2 years old, and the chance of clearance is greater than that of adults [47]. Studies have noted that the 0–19 y population has the lowest incidence among all age groups [48]; thus, we conducted statistical analysis by further age stratification of the children. The HC hospitalization rate in the 1–3 y group was the highest, reaching 42.11%, followed by the 4–6 y group with a rate of 36.84%. The onset of HC in children is insidious, and the symptoms are generally mild, which causes the number of HCV infections to be seriously underestimated [49]. CHC is also common in pediatric patients, and the possibility of spontaneous recovery from CHC is very low. However, the liver disease of CHC children is usually mild, and there is less evidence suggesting the development of fatal hepatitis and cirrhosis [50].

The results of this study showed that mild chronic HC was the main type of clinical manifestation in hospitalized children (57.89%), and acute anicteric patients accounted for 31.58% of cases. The median LOS of all hospitalized children was 4 days, which is consistent with the clinical characteristics of HCV infection. In addition, 1 out of 19 children with HC had myocardial damage, and HCV involvement in the development of myocarditis has also been reported previously [51]. In terms of geographical distribution, it was noted that the high-prevalence areas of HCV are concentrated in central, south, northwest, and northeast China [52]. We found that the reported hospitalized cases of HCV were also mainly distributed in northwest (47.37%) and central China (36.84%). Currently, there are no safe and effective vaccines or antiviral drugs against HCV available for children [53, 54]. In particular, the most significant hurdle to develop an HCV vaccine is the high mutation rate of the virus, facilitating its ability to escape the host immune response [55].

There are some limitations to this study. 1) Although the FUTURE database contains FSMRs from 27 children’s hospitals distributed in seven geographic regions of China, here we only retrieved and report the cases as a result of infection with any virus within the family *Flaviviridae*, and the hospital admissions induced by imported or indigenous infection with Zika virus, tick-borne encephalitis virus, and West Nile virus have not been recorded in the FUTURE database in the past five years. Moreover, although there are indigenous and imported JE and dengue cases, we failed to confirm the etiological types of 454 JE cases and 14 dengue cases via FSMRs. 2) Given that most cases of flavivirus infection (especially JEV and DENV) are asymptomatic or exhibit a mild clinical manifestation with an imperceptible onset, most patients are usually treated during an outpatient procedure. Therefore, the number of reported hospitalized cases in this study should theoretically be much lower than the actual number of infected cases. Thus, the data only represent the real-world situation of some hospitalized patients. However, given the rapid development of the FRCPD monitoring system and the FUTURE database, the influence of this bias on future research will be gradually weakened. 3) JE and YF are vaccine-preventable diseases, but FSMRs do not record the vaccination history of hospitalized children. 4) The burden of hospitalization is disproportionately shared across regions of China due to perceived socioeconomic disparity. 5) Data on the long-term follow-up of inpatients were not collected.

## Conclusion

In summary, we combed through the FUTURE database, which includes data from 27 children’s hospitals in China, and described the general epidemiological characteristics and burden of hospitalized children infected by any virus within the family *Flaviviridae* (JEV, DENV, YFV and HCV). This information will help to better understand the spatial and temporal distribution of flavivirus infections and aid in the timely detection and prevention of indigenous transmission and outbreaks. The findings are also conducive to formulating targeted prevention and control strategies and monitoring, optimizing the allocation of prevention and control resources, and implementing effective public health prevention and control measures in China.
